# Emerging Trends of Nanomedicines in the Management of Prostate Cancer: Perspectives and Potential Applications

**DOI:** 10.3390/pharmaceutics16030297

**Published:** 2024-02-20

**Authors:** Rohitas Deshmukh, Vaibhav Singh, Ranjit K. Harwansh, Rutvi Agrawal, Akash Garg, Sudarshan Singh, Gehan M. Elossaily, Mohd Nazam Ansari, Nemat Ali, Bhupendra G. Prajapati

**Affiliations:** 1 Institute of Pharmaceutical Research, GLA University, Mathura 281406, India; vaibhavsinghcsc@gmail.com (V.S.); harwanshranjeet@gmail.com (R.K.H.); 2 Rajiv Academy for Pharmacy, Mathura 281001, India; agrawalrutvi96@gmail.com (R.A.); akashgarg983@gmail.com (A.G.); 3 Office of Research Administration, Chiang Mai University, Chiang Mai 50200, Thailand; sudarshansingh83@hotmail.com; 4 Faculty of Pharmacy, Chiang Mai University, Chiang Mai 50200, Thailand; 5 Department of Basic Medical Sciences, College of Medicine, AlMaarefa University, P.O. Box 71666, Riyadh 11597, Saudi Arabia; jabdelmenam@mcst.edu.sa; 6 Department of Pharmacology and Toxicology, College of Pharmacy, Prince Sattam Bin Abdulaziz University, Alkharj 11942, Saudi Arabia; nazam.ansari@gmail.com; 7 Department of Pharmacology and Toxicology, College of Pharmacy, King Saud University, P.O. Box 2457, Riyadh 11451, Saudi Arabia; nali1@ksu.edu.sa; 8 Shree S. K. Patel College of Pharmaceutical Education and Research, Ganpat University, Mehsana 384012, India

**Keywords:** prostate cancer, nanomedicine, nanoparticles, carbon nanotubes, cancer

## Abstract

Prostate cancer is one of the most life-threatening disorders that occur in males. It has now become the third most common disease all over the world, and emerging cases and spiking mortality rates are becoming more challenging day by day. Several approaches have been used to treat prostate cancer, including surgery, radiation therapy, chemotherapy, etc. These are painful and invasive ways of treatment. Primarily, chemotherapy has been associated with numerous drawbacks restricting its further application. The majority of prostate cancers have the potential to become castration-resistant. Prostate cancer cells exhibit resistance to chemotherapy, resistance to radiation, ADT (androgen-deprivation therapy) resistance, and immune stiffness as a result of activating tumor-promoting signaling pathways and developing resistance to various treatment modalities. Nanomedicines such as liposomes, nanoparticles, branched dendrimers, carbon nanotubes, and quantum dots are promising disease management techniques in this context. Nanomedicines can target the drugs to the target site and enhance the drug’s action for a prolonged period. They may also increase the solubility and bioavailability of poorly soluble drugs. This review summarizes the current data on nanomedicines for the prevention and treatment of prostate cancer. Thus, nanomedicine is pioneering in disease management.

## 1. Introduction

**Figure 1:**
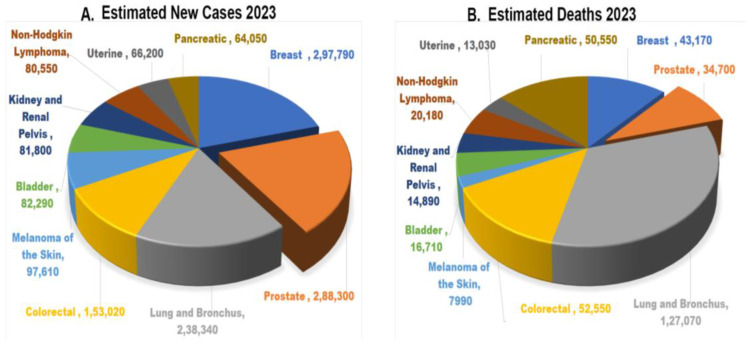
Cancer data: (**A**) Estimated number of new cases in 2023. (**B**) Estimated number of death cases in 2023 (data source: Cancer stat facts: Prostate cancer 2023).

Cancer is the main cause of most of the deaths in both developing and developed nations like India, China, the United States (U.S.), Africa, and many more. Prostate cancer (PC) is the most prevalent among different types of cancers, causing the highest number of deaths in men even after introducing advanced treatments and diagnostic methods. According to the latest research by Globocan and IARC (International Agency for Research on Cancer), the expected new cancer cases are 10,065,305 worldwide. The details of overall cancer cases estimated in the year 2023 (data source: Cancer stat facts: Prostate cancer 2023) are shown in Figure 1. In India, about 34,540 new cases of prostate cancer have been detected, which included the death of 16,783 men. About 115,426 and 106,139 cases have been detected in China and Japan, respectively. The reports revealed that around 51,094 deaths were reported in China and 13,426 in Japan. These data were used to predict the countries’ prostate cancer stats and its possible prevention and treatment prospects. PC is one of the fatal and most common cancers worldwide, especially in Americans. After lung and colorectal cancer, prostate cancer is the third most fatal cancer associated with death in Americans. PC appears mainly in older men; 6 out of 10 cases are found in males with age 65 and older (https://www.cancer.org/cancer (accessed on 9 February 2024)). PC is characterized by uncontrolled cell division, which leads the prostate to develop abnormally. Prostate cancer does not often kill men; instead, slow-growing tumors do. The metastasis of cancer cells to other bodily regions, such as the bone, pelvis, bladder, lumbar vertebra, rectum, and brain, is the primary cause of prostate cancer mortality [1,2]. There are different significant risk factors for PC, like age, smoking, diet, family history and genetic factors, and medications [2]. Age is firmly linked with the incidence of PC. There are four primary methods for the treatment of PC: chemotherapy, radical prostatectomy, androgen deprivation, and radiation [3].

Several anticancer medications are injected via systemic infusion to treat prostate cancer. Based on the method of administration and the area of the body covered, systemic and regional delivery techniques are two chemotherapeutic delivery mechanisms. Medications administered via systemic (infusion) delivery have significant disadvantages because of the body’s nonspecific distribution of the drugs. Acute problems and systemic toxicity are brought about by this nonspecificity, which causes the death of highly proliferative normal cells like gonads, bone marrow, GI mucosa hair, and follicles. Additionally, nonspecific drug uptake by healthy cells lowers the amount of medicine supplied to the intended malignant cells. To achieve therapy success, greater doses of cytotoxic medications must be given systemically. Cancer can be efficiently treated using traditional methods such as radiation and chemotherapy, whether used alone or in combination. A more intensive strategy is urgently required, though, to improve therapeutic effectiveness and lessen unfavorable side effects. Nanomedicines is one such approach for improved effectiveness and diagnosis. Nanotechnology-based medicines provide prolonged drug release, targeting effects, overcoming biological barriers, increasing solubility and bioavailability, etc., [4]. Drug-loaded nanoparticles, or nanomedicine, have been effectively used to actively target cancer at several levels of a tumor’s macroscopic structure and microscopic components by functionalizing a wide range of organic synthetic compounds, ligands, including oligopeptides, hormones, aptamers, antibodies, and vitamins. These methods have optimized the pharmacokinetic and biodistribution of nanomedicine, enhancing the effectiveness of the prescribed treatment through targeted drug delivery [5,6,7,8,9,10].

The main goal of prostate locoregional therapy is to give high medication dosages to the damaged organ or tissue. It would be hazardous for healthy tissues, cells, or organs when given systemically. Due to the apparent ease of method and operation, there needs to be more focus on using nanotechnology for locoregional prostate chemotherapy. However, in the recent past, much research has been carried out for treating diseases related to the prostate, such as PC and benign hyperplasia, using a nanotechnological approach. New approaches to nanomedicines, like liposomes, branched dendrimers, carbon nanotubes, and quantum dots, are also being utilized for prostate cancer. The present review points out various drug delivery methods that are utilized or are being researched to deliver nanomedicines in both preclinical models and clinical subjects.

## 2. Pathophysiology of Prostate Cancer

**Figure 2:**
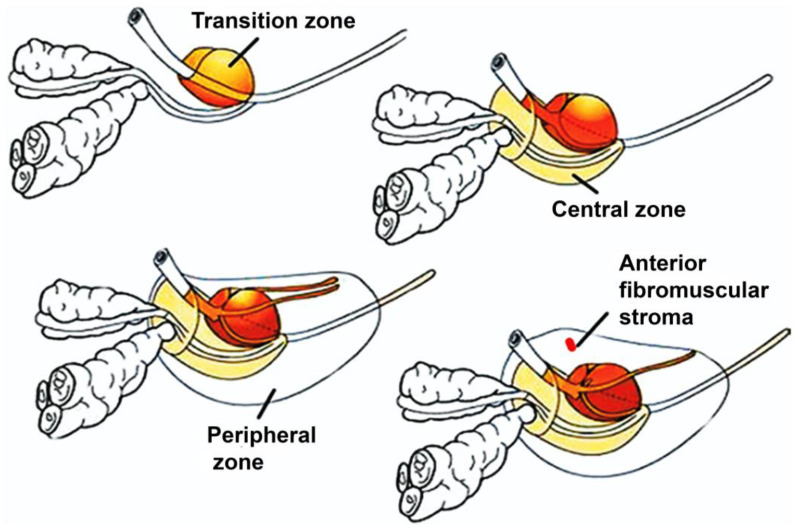
Various zones of prostate cancer. Adapted from [15] with permission.

The reproductive system of men includes the prostate gland, which aids in generating and storing seminal fluid. A typical prostate in adult males measures 3 cm in length and weighs 20 g [11]. Many small glands in the prostrate secrete 20% of the fluid that makes up semen [12]. PC is categorized into three zones [13]: central, peripheral, and transition (Figure 2). In a young adult, the transition zone makes up about 5–10% of the glandular prostate’s mass and surrounds the urethra at the point where the ejaculatory ducts are secreted. The central zone makes up about 20–25% of the glandular prostate’s mass and is below the proximal urethral portion [13]. The peripheral zone, a dual column of duct floret that laterally encircles the central zone and occupies the apical portion of the prostate, makes up about 70–75% of the normal glandular system of the adult prostate. The anterior fibromuscular stroma, which develop around one-third of the mass within prostatic capsules, is nonglandular [13]. About 70% of PC cases start in the peripheral zone [13,14]. The transitional and central zones account for 10–15% and 15–20% of prostate cancer cases, respectively [14,15].

## 3. Beginning and Progression of the Prostate Cancer Process

Prostate cancer is treatable initially until confined to the prostatic capsule. Since most instances of prostate carcinoma are indolent, most of the men who have received prostate treatment have died for other reasons. However, if not traced early, prostate cancer accrues a more aggressive form of the disease. Its local invasion in seminal vesicles, followed by malignancy to the bone, often results in mortality. Androgen-ablation treatment is one of the solutions for the shift from androgen dependency to androgen independence that follows this progressive sickness.

**Figure 3:**
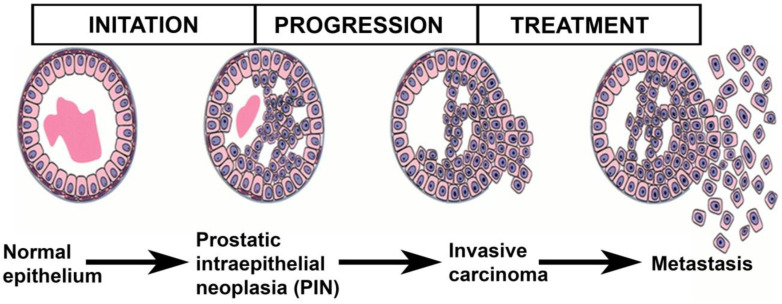
Progression of human prostate cancer pathway. Adapted from [16] with permission.

In contrast, research on the molecular level of prostate cancer growth and the contributors of prostate cancer has received comparatively less attention. Chromosomal changes common in prostate cancer were examined in the investigation. A fruitful area is the typical patterns of chromosomal aberrations in prostate carcinoma that are preferentially considered as a starting point for discussion of a progression route since they are indicative of the phases of prostate cancer advancement [16] (Figure 3). The decrease in potential tumor inhibition genes in prostate cancer is likely to be reflected in patterns of persistent allelic loss. Heterozygotic losses are most frequent on chromosomes 13q, 8p, 10q, and 17p. Losses of 16q, 6q, 7q, and 18q have also been documented, albeit less well defined. Additionally, gains at chromosomes 8q and 7 are highly prevalent, even though they occur less often than chromosomal losses.

Although allelic loss is essential for prostate malignant, no presumed tumor-specific gene has been given a function in cancer development. Based on their location in areas of allelic loss and their functional features of being altered in a significant portion of prostate cancer cases, many determinative applicant genes (e.g., RB, PTEN, p53, NKX3.1) have been identified. Unfortunately, the literature often contains contradictory findings on the incidence and kind of mutations in specific candidate genes. Therefore, there are several broad hypotheses to be taken into consideration for individual potential tumor-applicant genes. The first and most obvious assumption is that the cancer-fighting genes in the areas of allelic loss have yet to be found. It might be hard to find mutations in candidate genes because it is hard to obtain samples of tissue that are not too mixed up. This is because tumors are often different and have many other parts. Therefore, most tests are still conducted on huge tissue samples that are unlikely to be homogenous. However, this technical explanation is reasonably sound.

Real-time PCR, for example, allows for the study of a small number of cells, which may assist with these challenges. New advancements in PCR technology may also provide aid. Thirdly, candidate genes can be rendered inactive by a method other than a mutation in the coding area. Examples of such mechanisms include promoter methylation or mutations in regulatory regions that might impact translation, transcription, or mRNA stability. Additionally, inactivation might result from changes in a regulatory pathway’s upstream or downstream elements. Only a few studies have simultaneously examined numerous aspects of a particular route. The last idea is that haplo-insufficiency (loss of a single allele) may be crucial in the development of PC, which would align with the fact that most tumors have an indolent character and advance slowly like phenotype [16].

## 4. Risk Factors of Prostate Cancer

**Figure 4:**
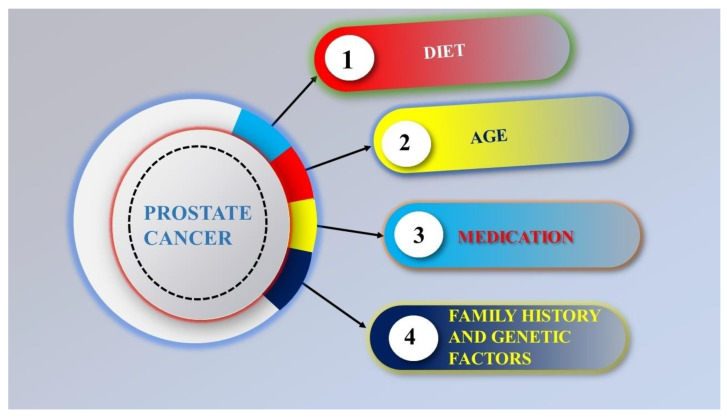
Risk factors of prostate cancer.

The causes of prostate cancer are still not fully understood [17]. Obesity, age, genetic factors, medication, and family medical history are the primary causes of prostate cancer (Figure 4). In males under 45, prostate cancer is very rare, but as men age, it becomes common. The average age at the time of treatment is 70 [18]. Inactivity, hyperglycemia, inflammation, infections, chemical environment, exposure or ionizing radiation, and diet (small intake of fruits, enhanced usage of saturated animal fat and red meat, vegetables, coffee, and vitamins) are additional factors that are attached to prostate cancer [19].

### 4.1. Age

Prostate cancer is the most dangerous disease in old-aged males [20]. The age–incidence curve for prostate cancer is the steepest of all illnesses, with a sharp rise in the seventh decade [21,22]. It has been noted that among white males without a family history of PC, the risk grows significantly after the age of 50 who are over 40, black males, or men with an antiquity of prostate cancer in their families, and who have no personal or family history of the disease [23]. Prostate cancer progression is slow, and older men with other serious health problems are likelier to die from other problems than prostate cancer.

### 4.2. Diet

Dietary elements might significantly impact PC growth, as shown by various research on immigrants migrating from low-risk locations in poor nations to high-risk areas in industrialized countries, demonstrating how the transition to a “Westernized” lifestyle caused an increase in the incidence of prostate cancer. For example, according to Chu et al., the PC rate in Americans was 40 times higher than in Africa [24]. There is little evidence that eating fruits and vegetables affects the risk of developing PC [25,26]. Another study on the human population found that red meat had no relation with risk increment. In several research studies, eating more meat has been related to a greater risk [27]. Prostate cancer risk may be increased by low vitamin D levels in the blood [28,29].

### 4.3. Family History and Genetic Factors

PC dangers may be caused by genetic factors, which have been linked to specific gene variants, family, and race. Men who have first-degree relative members with prostate cancer (father or brother) are twice as likely to have a risk of prostate cancer themselves, and those with two afflicted close relatives are at five times greater risk than those without a family history [30]. Family history matters; however, only 35% of the familial risk is attributable to known genes [31,32].

### 4.4. Medication

There are some connections between prostate cancer and drugs in medical treatments and diseases. Utilizing cholesterol-lowering medications might decrease the chance of developing PC [33,34]. The synthesis of lipids is inhibited by adopting drugs proven to lower PSA levels [35]. Before radical prostatectomy, patients in one study were given either simvastatin or a placebo. The change in benign and cancerous tissue in the prostate specimen was then studied [36]. This study found that simvastatin did not have any positive effects on health and was linked to many harmful side effects (+55% vs. 18.75% in the control group). The various drugs that are under clinical trials are listed in Table 1.

## 5. Diagnosis

Although PC can be treated if diagnosed early, most cases are identified when the cancer has progressed to the lymphatic or bone systems, which results in a dismal prognosis [37]. Prostate cancer must be diagnosed as soon as possible in order to be treated. Traditionally, the diagnosis of prostate cancer involves tissue biopsy, rectal examination, and the identification of prostate-specific antigen (PSA) by routine biochemical methods [38,39]. The preliminary screening is achieved using the standard biochemical method. Subsequently, a digital rectal examination (DRE) is performed to examine the prostate gland, with particular attention to its size and texture [40]. Biopsies are performed following DRE and are also included in the traditional diagnostic approach [41]. All three have several limitations, even though they are all often used for PC identification. The PSA’s sensitivity and specificity are incredibly low [42].

Similarly, DRE cannot offer early PC identification, even in cases where the tumor gland status may be directly seen [43]. Furthermore, the complete detection procedure is exceedingly painful and unpleasant because it involves near contact with the prostate gland of the patient. One major disadvantage of biopsies is the possibility of infection by bacteria that is inflaming the patient’s gland [44]. Furthermore, the course of treatment for PC depends greatly on its stage, and none of these diagnostic techniques can identify PC at the preliminary stage [45].

Patients having PC are not diagnosed at the initial stage because of the limited specificity of the prostate-specific antigen. Therefore, extensive diagnostic platforms that might raise prostate cancer diagnosis accuracy are desperately needed. Dysregulated miRNAs have emerged as interesting diagnostic and prognostic indicators for PC and are intimately linked to the disease’s progression and recurrence. DNA-conjugated gold nanoprobes (DNA-AuNPs) were devised, manufactured, and proven to be a feasible method for the one-step measurement of miR-21/miR-141/miR-375 in a study by Kshirsagar et al. using training and validation sets, and correlation studies based on the receiver operating characteristic curve in human sera demonstrated a distinct ability to distinguish PC patients from healthy controls. They developed an integrative nanobiosensing technology for various miRNA detection in liquid biopsies that are noninvasive and PCR-free for prostate cancer diagnosis [46].

## 6. Old Treatment Strategies for Prostate Cancer

Immunotherapy is a convenient option for diagnosing PC [47]. Various radiation therapies [48], chemotherapy [49], salvage and adjuvant treatment [50], hormone treatment [51,52], positron emission tomography (PET) with prostate-specific membrane antigen (PSMA) [53], and focused ultrasound with high intensity are some of the widely accepted techniques [54,55]. Surgical tissue removal [56], nonsurgical methods [57], psychological assistance, psychological usage, and use of drugs are also options [58]. Generally, PC is treated with chemotherapeutic medicines: cyclophosphamide, paclitaxel, docetaxel, cabazitaxel, etc., [59]. Traditional chemotherapy treatments not only kill cancer cells that divide quickly, but they also harm healthy cells that generally divide quickly, such as cells in the macrophages, bone marrow, hair, and digestive system [60]. Despondently, conventional chemotherapy drugs have many difficulties, such as low bioavailability, less circulation time, and increased drug resistance, which influence the accelerated division of healthy cells and chemotherapy-specific adverse effects (hepatic, cardiac, bone marrow, and renal toxicity). These significantly negatively impact the patient’s quality of life [61]. Immunotherapy causes dryness, blistering, and skin response. In certain cases, side effects such as flu-like sickness, dizziness, fever, weakness, nausea, high blood pressure, and chills are often seen in patients who undergo immunotherapy [62]. Additionally, radiation treatment has extra drawbacks, such as target restriction, immobilization of the patient, high expense, time commitment, and complexity [63].

## 7. Prostate Cancer Treatment by Novel Drug Delivery System

**Figure 5:**
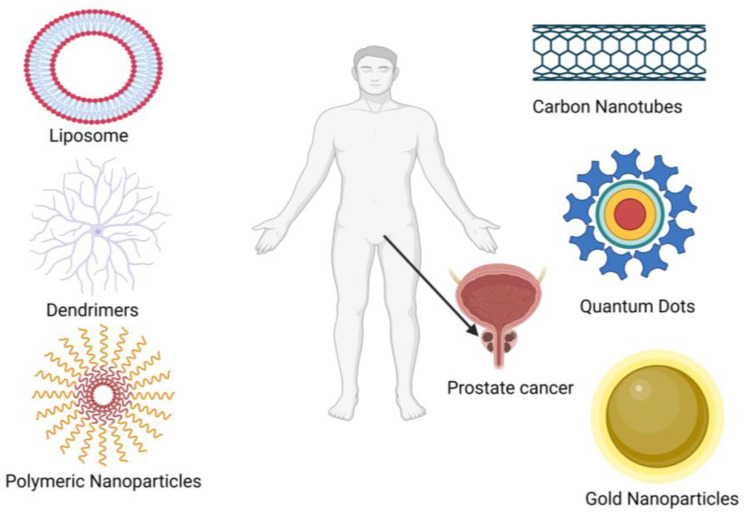
Treatment of prostate cancer using novel methods of drug delivery. The figure was redrawn through creative common attributes.

Nanodrug delivery methods are highly needed in cancer treatment and have undergone substantial research, as shown in Figure 5. Both organic (polymeric nanocapsules, niosomes, liposomes, and nanoemulsion) and inorganic (gold nanoparticles, quantum dots etc.) nanocarriers have shown excellent effectiveness as drug delivery vehicles for actively targeting prostate cancer [64]. Various research studies are ongoing regarding nanocarriers containing therapeutic agents for the treatment of prostate cancer, which are listed in Table 2.

### 7.1. Nanoparticles

Colloidal particles ranging from 10 to 1000 nm in size are known as nanoparticles (NPs) [65]. NPs have become more popular and diverse in the last few years due to their usefulness, prolonged shelf life, enhanced drug-loading capacity, and ability to deliver hydrophilic and lipophilic drugs through various routes [66]. They have been shown to make drugs less dangerous, concentrate drugs at disease sites, keep them in the body longer, protect them in the body, protect them the body, and protect drugs from humoral attacks [67]. Nanoparticles like metal nanomaterials [68,69,70,71,72,73,74,75,76,77,78], porous silicon nanoparticles, and liposomes [79,80,81] have been studied extensively to see how they can treat prostate cancer. Active targeting nanoparticles contain peptides, oligosaccharides, antibodies, or other molecules that have modified surfaces. Similar to the prostate-specific membrane antigen receptors on prostate cancer cells, these targeting devices target receptor cells on malignant cells [82].

Polymeric nanoparticles are colloidal systems with mostly 1–1000 nm diameters fabricated using biodegradable and biocompatible polymers [83]. They are divided into nanospheres (polymer matrices) and nanocapsules (polymeric walls with a core) [84,85].

Zachary et al. developed polyester copolymers using the standard melt polymerization method; these are polymers further modified into polymeric nanoparticles functionalized with folate for the targeted medication for prostate cancer and by using the prostate cancer cells model. Pseudo-branched polymers are spherical and employ the solvent diffusion approach to water-dispersible polymeric nanoparticles, delivering therapeutic doxorubicin (DOX) medication. They target PSMA receptor upregulation of LNCaP prostate cancer cells [86].

Fang et al. developed aptamer-coupled multifunctional polymeric nanoparticles for drug delivery. In order to diagnose castration-resistant prostate cancer, effective and cancer-targeted magnetic resonance imaging contrast agents are being developed and evaluated. Triblock (PLGA-b-PEG-b-Wy5a) self-assembly methods target the CPRC cell line pc3 utilizing the cell-SELEX approach [87].

Wang et al. synthesized a novel tumor acidity-activatable macromolecular nanoparticles-based drug as P-PDL1-CP, including a conjugated long-chain polyethylene glycol [88] encasing and a poly (2-diisopropylaminoethyl methacrylate) (PDPA) core. Long-chain PEG improved the tumor accumulation of the nanodrug by reducing the nonspecific cellular uptake and on-target off-tumor affinity of aPD-L1. Immunotherapy-related release of aPD-L1 was initiated by tumor acidity. In the meantime, the long-chain PEG was lost, and the remanent nanodrug’s internal charge switch improved PDPA absorption by cancer cells [89].

Alserihi et al. assessed the anticancer activity of Epigallocatechin gallate and Epigallocatechin gallate-loaded nanoparticles (EGCG NP) for the therapeutic management of PC in an in vitro three-dimensional spheroid model. After the administration of EGCG and EGCG nanoparticles, the spheroid diameters of the cell lines under investigation were substantially decreased. The mitochondrial membrane potential of 22Rv1 and PC-3 spheroids treated with EGCG and EGCG NP did not show any appreciable alterations. When compared to EGCG alone, the nanoform formulation demonstrated greater potential. Preclinical in vivo research and prospective nanoform modification should be performed to confirm the in vitro findings and increase the possibility of using it in clinical situations [90]. The study enhanced the therapeutic effect, which shows its potential for the treatment of prostate cancer in the future.

Wang et al. developed a dual-targeting approach using polylactide-polyethylene glycol-2-(3-((S)-5-amino-1-carboxypentyl)-ureido) pentanedioate/triphenylphosphonium (PLA-PEG-ACUPA/TPP) nanoparticles, which target mitochondria and PSMA in order to co-deliver this ratio of Ingenol-3-angelate (I3A) and DOX to tumors and guarantee their uptake. The delivery of these nanomedicines resulted in a potent antitumor immune response as well as reduced tumor development. It demonstrated that I3A blocked VEGF expression in tumor cells to restore normal tumor vasculature and induced mitophagy and death in human and mouse prostate cancer cells [91].

Tan et al. examined the anticancer effects of betulinic acid (BA), ceranib-2 (Cer), and the combined use of betulinic acid with ceranib-2 (BA-Cer) on PC-3 cells in vitro. In order to examine this, they first developed an appropriate nanocarrier utilizing a new Zn: MnO_2_ nanocomposite (NCs) and a polymeric shell consisting of gallic acid (GA), polylactic acid (PLA), and alginate that has good stability and nanoparticle size. The findings showed that free BA and Cer had no discernible cytotoxic effect on prostate cancer cells. Due to its magnetic properties, BA-Cer/Zn: MnO_2_@GA-PLA-alginate functions as a nanocarrier that is employed as a therapeutic agent as well as for imaging. It also has improved drug loading and release capability for hydrophobic medicines. Additionally, the combination of the drugs BA and Cer showed enormous potential in the treatment of PC [92]. This study depicts the potential theranostics applicability of developed nanoformulation for prostate cancer.

Piroxicam-entrapped hybrid nanocarriers were developed by Sengel-Turk et al., and their effectiveness against prostate cancer cells in vitro was assessed along with their nanoparticle characteristics. Studies on cell viability, apoptosis, and cell cycle arrest were used to assess the core–shell nanosystems in vitro using cell cultures. This study shows that, as compared to the pure molecule, the designed core–shell nanoparticulate formulation of piroxicam offers a more promising therapy option for prostate cancer [93].

Ghosh et al. demonstrated the development of a nanoparticle-based dual-mode nanocarrier for synergistic photodynamic therapy. Tetra carboxy zinc phthalocyanine, an ROS generator, and DOX, a chemotherapeutic drug, were successfully embedded into ZIF-8, a metal–organic framework. In comparison to DU145 cells, LNCaP cells absorbed the nanocomposite-coupled folic acid greater. It is interesting to note that, even at lower pH levels, covering nanocomposite using biocompatible polyethylene glycol greatly reduced the release of DOX. These findings imply that a polymer coating more closely regulates the release of DOX [94].

Different studies conducted by various researchers show the immense potential of nanoparticle-based formulations for the treatment of prostate cancer; however, more clinical evidence is necessary to prove their effectiveness over conventional therapy. The polymers and other functionalized carriers utilized need to be studied further.

### 7.2. Novel Drug Delivery System on the Basis of Ingredients

#### 7.2.1. Liposomes

Liposomes are the nanodrug delivery system that works well enough to be used in real-time clinical settings [95,96]. This idea changed the pharmaceutical field forever [97]. Alec Bangham [98] first talked about liposomes in 1961. Since then, there has been a lot of research on them. They are now employed in many areas, such as genes, drugs, and biomolecule delivery [97]. Compared to other DDNs, they have many benefits, such as easy dissolution, reduced systemic toxicity, and compatibility with the body [99]. A liposome is a versatile, self-assembling carrier material made up of one or more lipid bilayers [100]. The main lipid components of liposomes are cholesterol and phospholipids. Hydrophilic medications can be contained within the water cavities, while hydrophobic drugs are exposed to the lipid bilayers outside [101].

Zhang et al. devised a drug delivery method for the simultaneous administration of resveratrol (Res) and Docetaxel (Doc) using a liposomal system. The results showed that both drugs could be sent to the PC-3 cells, where they are released slowly from liposomes. In animal studies, PC-3-bearing nude mice given Doc/Res with liposomes were less toxic and lived longer than control mice given Doc/Res without liposomes [102]. Additionally, castration-resistant prostate cancer has received less attention in this research since castration-resistant PC is the subject of the sparsest studies focusing on the efficacy of liposomes in PC therapy. Clinical trials of prostate cancer have only been carried out with liposome DOX, an advanced cancer that is lethal and for which there are currently few viable therapies [103]. The study results show that liposomes encapsulating drugs were less toxic effects than drugs without liposomes. Further clinical studies should be carried out using liposomes encapsulating both drugs.

Lu Zang et al. designed a PEGylated nanoliposome for the combined delivery of resveratrol and docetaxel to increase the antitumor effect of the combination medicine used to treat prostate cancer. Where liposomes with cellular absorption might efficiently transport payload into cells, Doc/Res co-loaded liposomes have shown a significant increase in cell toxicity of both the medications on cancer cells, with a molar ratio of 1:2. In the investigation of caspase 3, the co-encapsulation of docetaxel and resveratrol in liposomes significantly reduced tumors development in pc3-bearing Balb/C nude mice, and changes in cell proliferation and apoptotic parameters were also developed. In this work, Dox/Res loading in a nanoliposome synergistically treats prostate cancer [102]. This study shows better results and provides clinical evidence of the beneficial effects of Res- and Doc-loaded liposomes in prostate cancer.

Ning et al. developed herceptin-coated liposomes co-loaded with DOX and simvastatin to diagnose PCA. In this study, a combination of the drugs simvastatin and DOX drug-loaded nanocarriers with antibodies that target receptors that are overexpressed on tumor cells was guaranteed to make chemotherapies less harmful for normal tissues. In the pc-3 cell lines, a combined delivery system coated with Herceptin for PCA underwent a physiochemical examination for cytotoxicity absorption, as human epidermal growth factor-2 is highly expressed and constitutively active. Dox and Sim, with an average particle size of 134 nm, are 80% more effective in delivering drugs inside liposomes. Their synergistic antiangiogenesis makes Dox and Sim work simultaneously against tumors. Therefore, these are the combination for strong suppression of PCA in vivo and in vitro with the inhibitory rate of tumor volumes equivalent to 36.80% and 68.70% of Herceptin-coated liposomes [104].

**Figure 6:**
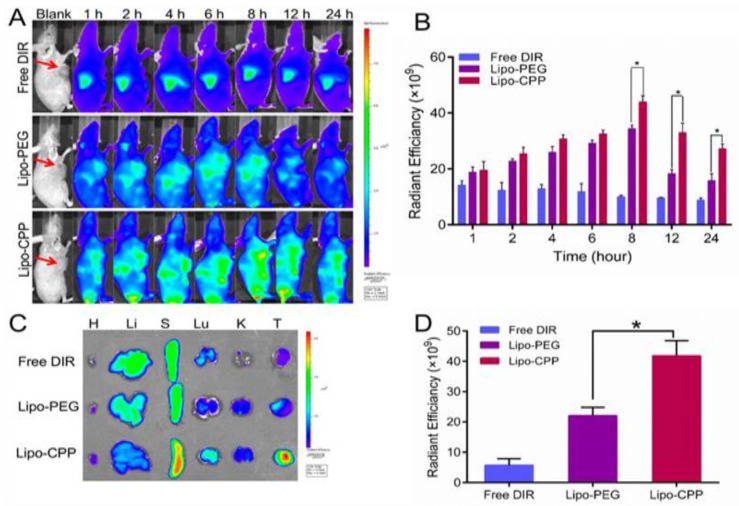
Liposome biodistribution in a tumor model. (**A**) Whole body imaging for 24 h. (**B**) Tumor sites showing in vivo radiant efficiency. (**C**) Ex vivo imaging of the vital organs eradicated from the mice. (**D**) Radiant capability of the tumors ex vivo. * represents levels of significance having *p*-value is less than 0.05. Adapted from [105] with permission.

The cellular uptake results suggest using Herceptin as a target receptor to place DOX and Sim into prostate cancer cells. The effectiveness of liposomes in targeting tumors in vivo was examined [104]. Nude mice with PC3 human prostate cancer xenografts were developed so that the biodistribution and tumor selectivity of liposomes with or without Herceptin modification could be measured. DIR was used as the model medication; within an hour of being injected intravenously into a mouse’s tail vein, the fluorescence of the tumor was observed. After that, the fluorescent intensity at the tumor site progressively spiked and peaked highest after eight hours. Real-time in vivo imaging revealed that the H-Lip group had greater fluorescence intensity at the tumor location than the Lip group (Figure 6A,B). PEG-modified liposomes have a long half-life and mean residence time, which indicates that they may remain in tumors for a very long duration. Animals were killed immediately after the in vivo imaging. The main organs and tumors were taken out, and images were obtained. Imaging of the dissected tissues revealed that the H-Lip group had more tumor accumulation (Figure 6) [105].

For treating advanced-stage PC, Quick et al. determined the application of RNA interference (RNAi) to target a region that is common across all androgen receptor splice variants for breakage and destruction. The results showed that in 22Rv1 and LNCaP cells, which depict two distinct prostate cancer models, 2′O methyl alteration of the siRNA (siARv^m^) increased androgen receptor (AR) and AR-V7 mRNA silencing potency. Researchers designed siARv^m^-LNPs for in vivo delivery. When compared to siARfl-LNP and siLUC-LNP control, therapy with siARv^m^-LNP significantly suppressed the growth of tumors and enhanced survival in mice carrying 22Rv1 tumors [106].

A novel approach for treating advanced prostate cancer by addressing nonspecific cleavage of the prostate-specific antigen-activable prodrug DOX (L-377,202) was explored. To avoid systemic activation, in response to mild hyperthermia, Pereira et al. enclosed it in low-temperature-sensitive liposomes (LTSL), which preserved its biological function upon controlled release. For both solid and metastatic prostate cancer tumor models, Dox-PSA-loaded LTSL slowed tumor growth at a pace comparable to mice subjected to free Dox-PSA. This suggests that this approach might prevent Dox-PSA’s systemic cleavage without lessening its in vivo effectiveness [107].

The above studies show the application of various drug moieties loaded into liposomes in decreasing the volume of tumors, preventing tumor progression, and detecting prostate-specific antigens. The liposomes target prostate cancer cells, specifically preventing noncancer cells from damage.

#### 7.2.2. Carbon Nanotubes

CNTs are tiny structures fabricated up of carbon and graphite. They are well-organized, hollow, and have many different properties. Some have a large surface area, and some are lightweight. CNTs are often placed into two groups: single-walled (SWCNT) and multiwalled (MWCNT). Single-walled CNTs are made of a single tube-shaped carbon sheet with a diameter between 0.4 and 2 nm, depending on how hot they were when formulated [108].

Li et al. studied carbon nanotubes, which include siRNA delivery for site-specific delivery of active substances [109]. In another study, CNTs were coupled and functionalized with amine 1, 2-stearoyl-sn-glycerol-3-phosphoethanolamine-N-(amino (polyethylene glycol) DSPE-PEG 2000 poly ethylenimine and maleimide for target and then conjugated with peptide (Asn-Gly-Arg) [110]. It was better at penetrating the human PCa-3 membrane in vitro, which makes it harder for cells to multiply and causes them to die off more quickly. Another RNAi-RNA photothermal treatment combined with no additional adverse side effects improved the antitumor effectiveness [111].

Fenfen Gu et al. developed a nanoultrasound contrast agent by adding polyethylene glycol [88] with multiwalled carbon nanotubes (MWCTNTS) to improve them into more water-soluble, stable, and anti-PSMA aptamers. Comparing modified MWCNTS to conventional contract agents, the latter could target Pca cells more successfully. The in vitro cytological study showed that the new contract agent worked well. Both in vivo and in vitro ultrasound imaging depicted that the new nanoultrasound contracting agent had a better effect on cell development, metabolism, and distribution. It may be a better-targeted ultrasound contracting agent for PCA [112].

Leila Farzin et al. developed ultrasensitive recognition of prostate-specific antigens. Multiwalled carbon nanotube (MWCNT)/L-histidine is lowered graphene oxide (His-rGO), which is shown to be a bifunctional nanoplatform for covalently connecting the thionine redox marker and anti-PSA antibody (Ab). The MWCNT improved the electricity flow and made it easier for electrons to move from the glassy carbon electrode to the thionine. Anti-PSA antibodies prevented thionine from transferring electrons, which reduced redox signals. The suggested immunosensor’s response was based on thionine-NH2-GO-COOH-specific Ab’s interaction with PSA. The response current of the linked electrochemical probe was further reduced due to this selective contact. The immunosensor found PSA in human blood and saliva samples, which suggests that the proposed method could be utilized to check the status of tumor markers in patients with PC [112].

Carbon nanotubes have theranostics application in prostate cancer. The functionalization of carbon nanotubes provides targeted delivery to prostate cancer cells. The multiwalled carbon nanotubes coupled with various moieties work as better contrast agents and help in studying progression of tumor in patients.

#### 7.2.3. Quantum Dots

Quantum dots (QD) are used for PCa-focused delivery. Their size is between 2 and 100 nm. They consist of semiconductor elements with particular photoluminescent and electrical properties from periodic groups II to VI or III to V [113]. The optical properties of QD come from how they are fabricated. The same substance is used as a coating or shell layer to cover the core of semiconductor materials like lead selenide, cadmium selenide, and indium arsenide [114]. Cell behavior may be tracked using nanotechnology based on QDs (like motility, adhesion, invasion) and track therapy responses in vitro and in vivo, presenting new opportunities to doctors and patients to diagnose and treat cancer [115]. QD comprises crystalline metalloids and has the effect of quantum limiting. In vivo studies show that passive and active targeting can be used to perform QD tests on cancer cells (retention effect and via increased permeability). To achieve an actively targeted distribution, antibody-legated QDs are used to target prostate-specific membrane antigens (PSMAs). In PCA treatment, PSMA was chosen as the main therapeutic and diagnostic target. The basis of PCA targeting and scanning is the retention and concentration of PSMA antibodies at the location of the cancerous cell [116].

Jigyasu et al. developed quantum dots containing cadmium telluride (CdTe). Yeast cells are used in a biological process to make quantum dots. Antiproliferation activity was determined using the MTT test. Using a flow cytometer, the production of reactive oxygen species (ROS), nuclear apoptosis, and the cell cycle was studied using a PC-3 cell line. The antiproliferative test in vitro showed that CdTe QDS killed cells dose-dependently and caused nuclear apoptosis. Additionally, CdTe grows cancer, which may offer insightful information for its clinical use [117].

Vuyeluva Ncapayi et al. developed near-infrared ternary AgInSe/ZnS quantum dots (QDS) for both bacteria and cancer. Quantum dots demonstrated minimal toxicity against mouse colon cancer (C26), mouse mammary carcinoma (Fm3A-Luc), cancerous fibrous histiocytoma-like (Km-Luc/GFP), and prostate tumor cells. *Staphylococcus aureus* had more significant accumulations, and prostate cancer cells took up QDS well. It is a big step towards using quantum dots to help diagnose and treat prostate cancer [118].

The studies show the development of quantum dots as an advanced approach for cancer treatment, but they need to be evaluated clinically for safety purposes.

#### 7.2.4. Dendrimers

The assumption of dendrimers was first talked about in 1985 [119]. In 1990, Frechet et al. developed a convergent method for making dendrimers [120]. Dendrimers are being studied as new nanostructures in prostate cancer at present. Dendrimers are nanoscale globular macromolecules that can be used to administer nanoformulations and circumnavigate the drawbacks of traditional treatments [121]. Nanodrugs developed from dendrimers can help find tumors early on and keep track of their characteristics, aggressiveness, spread, and prognosis [122]. In vitro, their highly branching 3D structure and different functional groups make it easier to catch cancer markers and improve the capture density per unit area [123]. In the case of tumor detection in living organisms (in vivo), their enhanced biocompatibility and low viscosity are the basis for in vivo applicability [124].

Yiwen Dong et al. developed amphiphilic phospholipid peptide dendrimers (AmPPDs) that might be utilized to deliver siRNA targeting Hsp27 for diagnosing castration-resistant PC. DSPE-KK2, AmPPDs with shorter hydrophilic dendron, promote more effective intracellular absorption and endosome release of the generated siRNA complexes, as well as stronger gene silencing and anticancer effects both in vitro and in vivo, due to increased siRNA releasing abilities [125].

The dendrimers can be used to target cancer cells and can be utilized for both diagnosis and therapeutic purposes. However, they need to be studied more for their mechanism and efficacy in the human body.

#### 7.2.5. Gold Nanoparticles

The sizes of gold nanoparticles (AuNPs), colloidal or clustered particles, vary from a few to hundreds of nanometers. AuNPs are composed of an Au core. AuNPs medicinal applications, including medication transport, radiation, and phototherapy, show versatility [72,126]. It is a valuable and desirable material for biological, biomedical, and imaging applications, such as intravenous contrast agents for imaging, pacemaker leads, and noninvasive lung and prostate cancer detection, due to its unique characteristics of being nontoxic, inert, and biocompatible [127].

**Figure 7:**
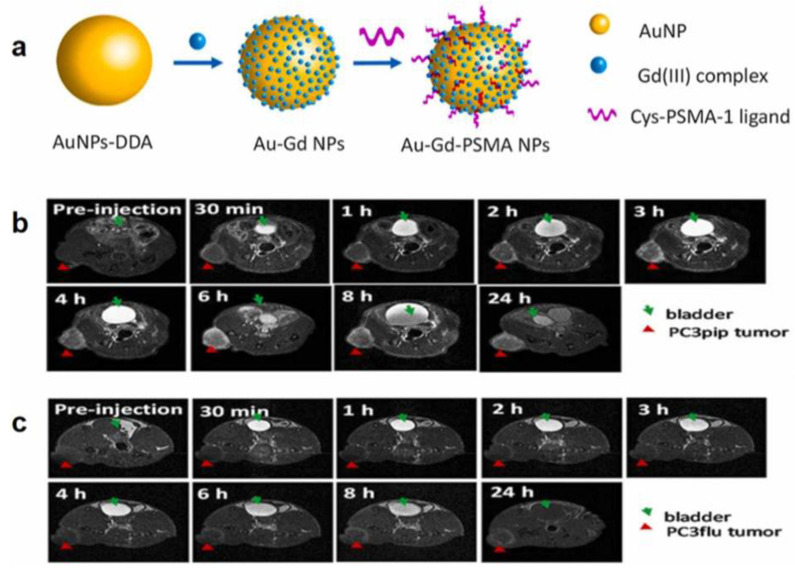
(**a**) Au-Gd(III)-PSMA NPs schematic representation for MR-guided radiation treatment. (**b**,**c**) NPs targeting tumors in vivo and MR imaging. Mice’s T1-weighted spin-echo images (**b**) PC3pip tumor and (**c**) PC3flu tumor were collected at 7 T. Tumors are indicated by red triangles, and bladders are indicated by green arrows. Adapted from [129] with permission.

Luo et al. fabricated AuNPs for MR-guided PC-targeted diagnosis by attaching gadolinium (Gd 3) compounds and PSMA-targeting ligands to the exterior of AuNPs. These changes to the surface made the r1 relaxation rate four times faster, which led to better binding capacity. The results showed that PSMA-expressing prostate cancer cells took up more gold nanoparticles and that MRI contrast was good in vitro and in vivo, and that gold and Gd binding [128] developed radiation better at stopping prostate cancer. It is proven that Au-Gd(III)-PSMA NPs are very selective for PSMA-expressing prostate cancer cells with better cellular MR contrast and in vitro radio sensitization (Figure 7). PSMA-targeted gold nanoparticles were able to go straight to the tumor, allowing accurate radiation treatment with less radiation and less damage to healthy tissues [129].

Docetaxel-encapsulated polyethylene glycol-functionalized AuNPs were developed by Thumbiraj et al. for specific medication delivery for prostate cancer. Docetaxel, an anticancer medication, was combined with AuNPs using noncovalent linking methodologies. Docetaxel-encapsulated AuNPs showed antitumor action in prostate cancer cells (Pc-3). The medicine docetaxel-encapsulated nanoformulation reduces cell viability, damaging prostate cancer cells following exposure to docetaxel-loaded AuNPs, which have cell toxicity action against Pc-3 cell lines for target drug delivery to PC [130].

For the targeted delivery, Baker et al. fabricated a combinatorial nanomedicine consisting of bioconjugated survivin-encapsulated gold nanoparticles (AbEzSvGNPs) conjugated with abiraterone and enzalutamide. AbEzSvGNPs were found to exhibit a synergistic effect of enzalutamide, abiraterone, and surviving encapsulated AuNPs against DU 145 and PC-3 cells. Furthermore, their effect was found to be greatly enhanced in comparison to the combined action of the drugs in the free form. Additionally, it was shown that AbEzSvGNPs were extremely safe and did not significantly harm the kidney cells of normal rats. The synthesized and assessed AbEzSvGNPs in the study indicate positive possibilities for PC treatment [131].

In order to precisely identify and measure PSMA expression in prostate cancer lesions, Wang MS et al. developed a new noninvasive MR/CT/NIRF multimodal contrast agent (AGGP) integrated with a high-affinity PSMA ligand (PSMA1). In living mice, these agents demonstrated powerful tripe-modal signal augments, preferential prostate-specific membrane antigen targeting, efficient mononuclear phagocyte system escaping, and profitable renal-clearable behavior. Research on biocompatibility and histopathology confirmed the high level of security of AGGP in vivo, providing potential avenues for future advancements in the early diagnosis of PC and the clinical application of more potent multifunctional nanotherapeutics [132].

Enzalutamide (Enz) was administered to the androgen-receptor-null cells, i.e., PC3 and DU145, by Baker et al., using enza bioconjugated survivin polyclonal antibodies-encapsulated gold NPs (EnSvGNPs). The delivery vehicle, SvGNPs, also has the potential to fight cancer and was found to work in association with Enz. Together with SvGNPs, the synergistic impact of enza was found to boost the efficacy. Through androgen receptor separate mechanisms, the propagation of DU145 and PC3 cells was successfully restricted by this innovative and ingenious targeted delivery approach [133].

The result of the studies shows the direct targeting effect of gold nanoparticles on damaged cells without harming healthy cells. Gold nanoparticles provide simultaneous delivery of two therapeutic agents, helping to enhance their efficacy in the treatment of prostate cancer.

#### 7.2.6. Micelles

Ni et al. investigated the impact of disulfide crosslinking on the effectiveness of integrin-targeting micellar docetaxel both in vitro and in vivo. The findings suggested that cyclic RGD and disulfide contents, in addition to tiny particle size, are important factors in defining t-MDTX’s in vivo activities, which include pharmacokinetics, stability, biodistribution, and active tumor-targeting potential. The enhanced efficacy of t-M5DTX is additionally associated with its targeting capabilities toward tumor necrosis and α_v_β_3_-overexpressing cancer cells [134]. Micelles, due to their small particle size, provide targeted delivery to tumor cells, which can be further enhanced by functionalization or cross-linking.

### 7.3. Novel Drug Delivery System on the Basis of Shape

#### 7.3.1. Nanorods

New, long, zinc oxide nanorods with a hexagonal cross-sectional configuration were successfully synthesized by Yadav et al. for the treatment of PC. Long and hexagonal zinc oxide nanorods have modest cell toxic effects on normal prostate epithelium RWPE-1 cells but can operate as a specific anticancer agent targeting prostate cancer cells, according to results of DNA fragmentation-induced apoptosis and additional in vitro cytotoxicity experiments on PC-3 cells. These findings showed that the zinc oxide nanorods have a dose- and time-dependent selective cytotoxicity effect on cancer cells [135]. The nanorods, being an effective nanosystem in treating prostate cancer, have mild toxic effects on normal cells of the epithelium. More studies need to be carried out and evaluated efficiently to prove their applicability.

#### 7.3.2. Nanosheets

Xu et al. studied the application of black phosphorus nanosheets in treating PC resistant to abiraterone. They found that the migration of these cells is greater than that of normal prostate cancer cells. According to the findings, black phosphorus nanosheets inhibited the migration and proliferation of cancer cells that are resistant to abiraterone. Gene ontology, enrichment analysis, and ingenious pathway analysis of the differentially expressed genes revealed that black phosphorus nanosheet treatment regulated the growth and metastasis of cancer cells as well as oncogenic signaling pathways [136]. Nanosheets have been found to be advantageous in the treatment of abiraterone-resistant cancer, which shows their potential in the treatment of prostate cancer. Their use needs to be researched more to evaluate their effectiveness and other potential benefits.

### 7.4. Others

In preclinical prostate cancer models, Vicente-Ruiz et al. investigated the effects of binding a Poly-L-glutamic acid (PGA) to an anti-Insulin growth factor-1 receptor antibody (AVE1642) on the bio–nano interface and antitumor activity. The results depict that PGA conjugation enhances AVE1642’s activity both in vivo and in vitro. The PGA conjugation modifies AVE1642 cellular trafficking, prevents AVE1642 from being degraded in serum, and increases AVE1642’s binding affinity to IGF-1R. These promising findings imply that PGA-AVE1642 may be a better therapeutic option than AVE1642 in prostate cancer [137].

Brachytherapy is a key effective treatment for prostate cancer that involves injecting seeds with radioactive substances directly into tissue. The primary drawbacks of this treatment are acute urine retention, sub-acute frequency and urgency of urination, and decreased effectiveness against severe tumor cells. Incorporating medicines into brachytherapy is one way to make it better. Hagan et al. used continuous liquid interface production to construct 3D-printed drug-loaded brachytherapy spacers with varying surface patterns to regulate drug release. Using a mouse model of prostate cancer, they investigated these spacers using dexamethasone and docetaxel as model medicines and discovered that the application of drug-loaded spacers increased the efficacy of brachytherapy and did not seem to result in any systemic risk [138].

Tumor microenvironment (TME)-activated nanoprobes were developed and assembled by Tan et al. In addition to acting as a TME trigger to induce drug release in response to TME, the CaCO_3_ shell could effectively captivate the photosensitizer IR820 along with the chemotherapy medication docetaxel (DTX) on the exterior of pentagonal gold prisms (PGPs) to inhibit removal from the circulation. This multifunctional nanoformulation using hyaluronic acid ((PGP/CaCO_3_@IR820/DTX-HA)) can meet the needs of NIR fluorescence imaging and photoacoustic imaging, as well as tumor-microenvironment-activated synergistic photodynamic therapy, photothermal therapy, and chemical therapy. Hyaluronic acid provides a unique tumor-targeting ability and biocompatibility [139].

**Table 2:** Nanocarriers containing therapeutic agents for the treatment of prostate cancer.

Nanocarrier	Key Ingredient	Therapeutic Agent	Targeting Agent	Reference
Gold nanoparticles	Prostate-specific membrane antigen	Au and Gd [128]	MR guided	[129]
Gold nanoparticles	Polyethylene glycol	Docetaxel	Folic acid	[130]
Gold nanoparticles	Bioactive phytochemicals	Resveratrol	PC-3 cancer	[140]
Polymeric nanoparticles	Polylactic-co-glycolic acid	Docetaxel	Wy5a-aptamer	[87]
Polymeric nanoparticles	Prostate-specific membrane antigen	Docetaxel	Folic acid	[86]
Dendrimer	Gold nanocages	Lactoferrin	DNA	[141]
Liposome	Nanoliposome	Doxirubicine and resveratral	Capspase-3 enzyme	[102]
Liposome	Herceptin	DOX and simvastatine	Human epidermal growth factor 2	[104]
Liposome	Polyethylenimine And polyethylene glycol	n-Butylidenephthalide	B16/F10	[142]
Carbon nanotubes	Polyethyleneimine	Paclitaxel	LnCaP	[143]
CdSe/ZnS quantum dots	Mesoporous silica nanoparticles	Paclitaxel	Prostate cancer cells PSMA^+^	[144]
Micelles	UniPR126	Niclosamide	PC-3 cells	[145]
Nanorods	Zinc oxide		PC-3 cells	[135]

## 8. Different Drug Delivery Routes for Targeting Prostate Cancer

**Figure 8:**
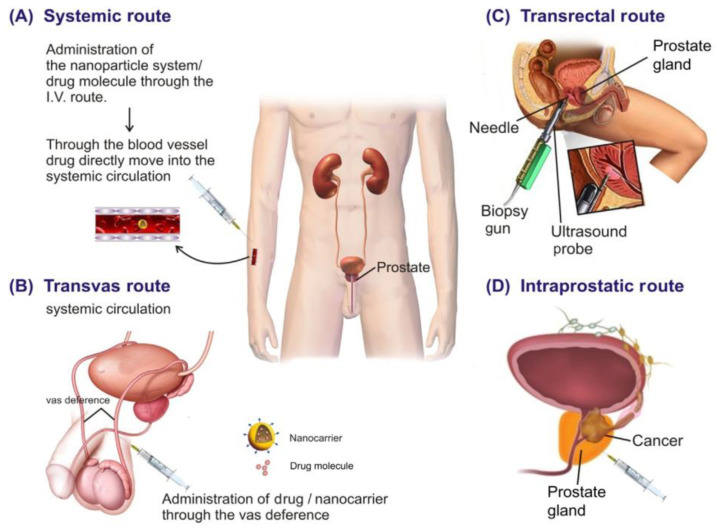
Various routes for targeting prostate cancer. Adapted from [146] with permission.

There are different types of routes for the delivery of therapeutics, which are depicted in Figure 8, and the drugs given by various routes are listed in Table 3.

### 8.1. Systemic Route

One of the biggest problems with studying PC is that scientists often use an animal model that does not precisely match how the human body functions because of differences in location, genes, and how genes and the environment interact. PC produced is orthotopic or subcutaneously (xenograft) with separate PC cell lines with or without being genetically altered. Although there may be matches in the pathogenicity of malignancy, there are still differences in the prostate’s physiological, hereditary, and neoplastic microenvironment in humans and animals [147]. In general, the systemic route of drug delivery and nanoparticles are often used to study prostate cancer in animal models covering various microenvironments. Various routes of drug administration, like intraperitoneal penetration [148], subcutaneous penetration [149], tail vein administration [150], retro-orbital administration [151], and i.v. [152], have been developed for treating PC. Because of the better penetration, the NPS and drugs move into the bloodstream and gather at the tumor site. Functionalizing tumor-specific ligands with NPs makes it easier for the NPs to reach the tumor site [82]. Table 3 demonstrates the multiple ways that are being investigated in preclinical studies for the treatment of PC.

**Table 3:** Description of different drug delivery routes for prostate cancer targeting.

Drug	Carrier	Targeting Moiety	Route of Administration	Dose	Animal Model	References
mRNA	Polymeric nanoparticles	Death -1	Tail vein	700 μg/kg	Orthotopic model	[153]
PTEN mRNA	Polymer lipid hybrid nanoparticles	Anti–HA antibody	Tail vein	700 μg/kg	Pc3 xenograft model	[154]
Docetaxel	PLGA nanoparticle	PCa	-	-	-	[155]
Curcumin and Cabazitaxel	Lipid polymer hybrid nanoparticles	A10-3.2 aptamer	Intraperitoneally	2 mg/kg–5 mg/kg	Xenograft model	[156]

Polymeric nanoparticles are utilized to actively target mRNA. The Phosphatase and Tensin homolog deleted on chromosome ten (PTEN) in mutated melanoma cells can be reintroduced into PTEN null prostate cancer cells in vitro and in vivo by encapsulating them in polymeric nanoparticles. This allows high PTEN mRNA transcription in prostate cancer cells and slowing of tumor growth in prostate cancer mouse models. In vivo, the medicinal use of PTEN mRNA nanoparticles in a PCA xenograft model was analyzed using the below experiment.

Six injections of PTEN mRNA PGDP nanoparticles were injected intravenously through the vein of the tail every three days in immune-compromised athymic nude mice bearing subcutaneous pc-3 xenograft tumor. It is a control for mice administered with PBS, and EGFP-mRNA-PGDP nanoparticles worked as control. Tumor development was observed to be the fastest in both groups. Nanoparticles showed reduced tumor growth compared to the growth in the control groups; tumor size rapidly increased to ~738 mm^3^ and ~674 mm^3^ for the mouse administered with PBS and EGFP mRNA PGDP nanoparticles, respectively, that are substantially larger than 288 mm^3^ for the group treated with PTEN-mRNA PGDP nanoparticles. The average tumor weight for the PTEN mRNA–PGDP nanoparticles treatment group is significantly lower than the control group. Nanodelivery is opted to invert the effect of tumor suppressor loss in prostate tumors. The in vivo therapeutic potential of PTEN-mRNA-PGDP nanoparticles makes HA-PTEN expression visible in tumors [153].

**Figure 9:**
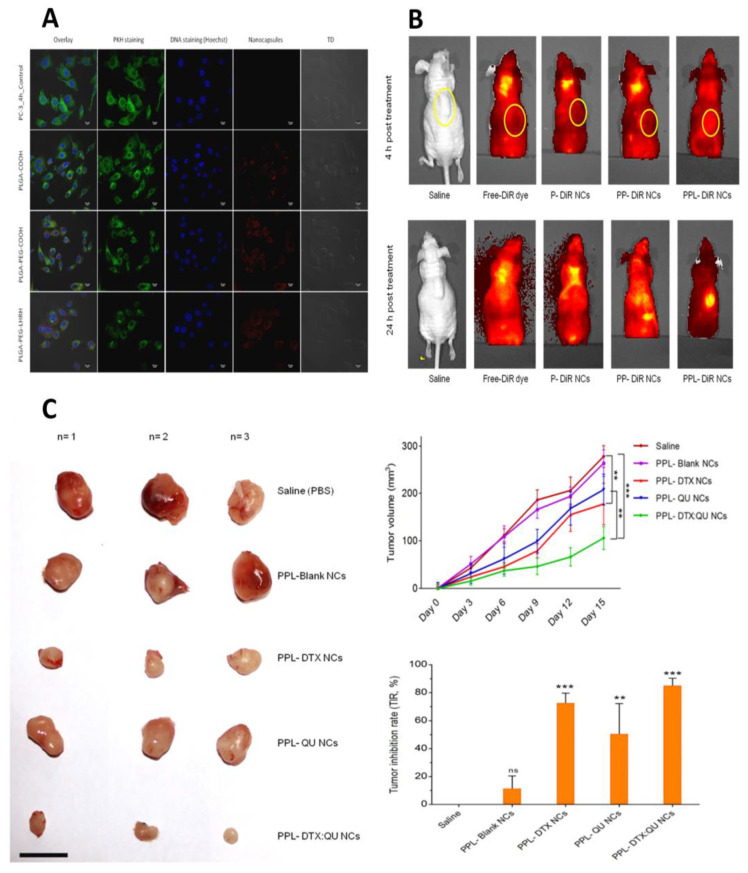
In vitro cellular uptake and in vivo antitumor activity of PLGA-PEG-LHRH NCs encapsulating combination of DTX and QU. (**A**) cellular uptake study of NCs in PC-3 cell line. (**B**) In vivo localization of NCs in PC-3 tumor-bearing Balb/c nude mice. (**C**) Antitumor activity of NCs in human prostate cancer bearing Balb/c, representing tumor size, tumor volume, and tumor inhibition ratio after in vivo administration, scale bar = 20 mm. Statistical significance: ** *p* < 0.01, *** *p* < 0.001. Adapted and modified from [157] with permission.

Ajinkya A. et al. developed chemically modified polymeric nanocapsules (NCs) to actively target prostate cancer (PCa) by encapsulating quercetin (QU) and docetaxel (DTX). By conjugating the Luteinizing-hormone-releasing hormone (LHRH) ligand to poly-lactide-co-glycolide (PLGA) and using polyethylene glycol [88] as a spacer, the active targeting was accomplished. The drug-encapsulated NCs had a negative zeta potential in the range of −20 to −40 mV and a homogeneous size distribution, with sizes ranging from 120 to 150 nm. The sustained drug release pattern from each NC was demonstrated by the in vitro release experiments. The LHRH-targeted PEGylated DTX: QU NCs displayed increased caspase-3 activity, and the LHRH-targeted NCs’ uptake was much higher, according to the results of the cellular uptake assays (Figure 9A). The entire animal was also monitored for in vivo imaging analysis at 4 and 24 h after the formulation, and the blank was injected intravenously (IV). The in vivo imaging data showed a greater accumulation of NCs at the tumor site after a 24 h therapy, as seen by a stronger fluorescence signal (Figure 9B). The reason behind this could be the size of the NCs and their tendency to gather at the tumor site due to the EPR effect. When dye-labeled PLGA-PEG-LHRH NCs were compared to other treatment groups, a sharper and greater fluorescence intensity signal at the tumor location indicated a comparatively higher tumor localization 24 h later.

Further, in an in vivo experiment, they demonstrated that tumor cells receiving saline or blank-NCs injections grow quickly. On the other hand, different treatment groups of drugs-encapsulated NCs showed varying degrees of anticancer efficacy. There was a notable decrease in the tumor volume in the PPL-DTX NCs and PPL-DTX: QU NCs groups as compared to the saline group (Figure 9C) [157].

### 8.2. Locoregional Routes

#### Intraprostatic Route

Intraprostatic drug delivery has been used to study preclinical and clinical trials. In preclinical models, much research has been carried out on drug-encapsulated implants and direct intraprostatic injection. In the real world, radioactive seed implants or brachytherapy have been used successfully [158]. The intraprostatic injection might be used to administer a large dose of medication with minimal side effects on the circulatory system [159]. They selected an animal model for an intraprostatic drug delivery investigation because of the living models’ physical and anatomical differences. Experimental models also play an essential role as the dog has approximately near similarities to the human prostate but some differences in the number of lobules, zone, shape, and secretion composition [160]. They are intraprostatic injections to treat prostate cancer in a specific area, but only a few drugs can be given [158].

Wieks Geiben et al. fabricated superparamagnetic iron oxide nanoparticles (SIONs) that target sentinel lymph node dissection. These nanoparticles are used to treat prostate cancer. Sentinel lymph nodes were detected by using magnetic resonance scanning and a magnetometer after intraprostatic injection of SIONs; a magnetometer was successfully given in PC. In order to assess the validity of this method, magnetometer-guided SLNP was analyzed in intermediate and high-risk PC patients, and on analysis, SLN was found in both in vivo and ex vivo tests [161].

### 8.3. Vas Deferens

Vas deferens are also recognized as the path that sperms use as they travel from the testis to the urethra [162]. This way of reaching out to the prostate with nanomedicines is exciting. In research, the compound styrene maleic anhydride (SMA) was used as an applicant drug system to show that it can deliver liposome-encapsulated drugs to the prostate gland longer [163]. In an investigation, the compound styrene maleic anhydride was used as a candidate drug model to show that it could be used to deliver liposome-encapsulated drugs to the prostate gland for an escalated time [164].

### 8.4. Transrectal Route

The rectum is one of the closest organs to the prostate [165], which makes it easier to diagnose diseases related to the prostate with an electronic rectal exam and transrectal UV prostate biopsies. Strangely, in addition to its use in diagnostics, ultrasonography has also been used in targeted nanodelivery methods for treating PC. This method of delivering nanoparticles through the transrectal route with the help of ultrasound is a potential way to treat PC [166]. Recent studies have shown that, in addition to its diagnostic contribution, ultrasound is one of the noninvasive ways to treat people. It has been shown that both high-frequency and low-frequency ultrasound can be helpful, with the low frequency being the best at entering tissues [167]. In people, secondary access to the prostate can be obtained most conveniently and least invasively through the rectal route.

## 9. Aptamers (Novel) Mediated Drug Delivery Systems

The aptamer was first discovered in 1990. Several groups of scientists used the binding property of aptamers to isolate a diversity of specific aptamers [168]. Aptamers are single-strand oligonucleotides ribonucleic acid (RNA) and oligonucleotides deoxyribonucleic acid [169] consisting of 20–60 nucleotides, having a three-dimensional structure that binds to the specificity with affinity similar antibody [170]. They offer less expensive production techniques and lower cost [171]. Aptamer-based nanosystems have been developed to deliver anticancer drugs to the tumor site to kill cancer cells and move deeper into the body. They deliver the drug to the tumor by acting as a molecular investigation to find and bind the conforming binding site. They are reactions between antibodies and antigens, which naturally change their three-dimensional moiety and help a receptor move the right place [172]. Aptamers have also been used to deliver nanoparticles to PC. Due to their specificity for target cells and fewer immunogenic reactions, aptamers have an advantage over other ligands. Also, the pharmaceutical sector is encouraged to use aptamer-based drug discovery since it is relatively simple to generate and assess aptamers for therapeutic as well as diagnostic purposes [173].

Yougian Fang et al. developed multifunctional polymeric nanoparticles that can control drug delivery to cancer cells and can be used for magnetic resonance imaging. They are core–shell targeted nanoparticles developed from PLGA and Wy5a aptamers that target the CRPC cell line pc3 cell using the SELEX technique. Targeted nanoparticles control drug release to enhance MRI capability. Wy5a and nanoparticles increase the cell toxicity of nanoparticles when delivered to PC3 cells with a cancer target in vitro and in vivo using MRI [87].

Yougan Chen et al. fabricated lipid polymer nanoparticles that can deliver curcumin and cabazitaxel together. At a drug ratio of 2:5, aptamer-functionalized curcumin and cabazitaxel lipid hybrid nanoparticles (APT-CUR/CTX-LPNs) demonstrated strong tumor accumulation, excellent cell inhibition capacity, and exceptional tumor inhibition efficiency. They use cancer cells and tumor xenograft in vivo models to double the number of drugs that reach the prostate. It is a prostate cancer synergistically combined treatment [156]. A particular protein kinase C-β inhibitor called enzastaurin considerably reduced neuroendocrine prostate cancer. Enzastaurin’s therapeutic applicability is severely limited because it has been shown to induce thrombus and other adverse effects in a number of clinical trials for different illnesses. Qie et al. fabricated a thrombin-binding aptamer and a functional polymer nanoparticle altered with SSTR2-targeted octreotide to deliver enzastaurin for neuroendocrine prostate cancer treatment in order to solve these problems. According to in vitro and in vivo results, the specially formulated nanomedicine effectively inhibited the growth of tumors and the frequency of coagulation disorders [174].

## 10. Future Prospect

As treatments for prostate cancer improve, they open up more ways to carry out research. NPs can be given in a general way, or they can be given in a specific way, like through the vas deferens or transrectally. Both of these methods have their problems. Also, some fields are just starting up, such as using aptamers for nanodelivery. In addition, the clinical trials of different NPs, such as gold NP, have made it possible to learn more about this area. In short, PC therapy needs to learn much more about the science of nanodelivery to understand the challenges and opportunities ahead.

## 11. Conclusions

Prostate cancer, being the most common cancer in men, needs a more precise way to deliver drugs that uses nanotechnology’s technological advances. The traditional method of using nanoparticles to deliver drugs to treat prostate cancer must be increased to obtain the best therapeutic results with the fewest side effects. Interestingly, the selectivity of NP-based drug delivery could be improved by engineering the surface of specific nanoparticles. However, choosing a suitable surface marker is crucial for targeted NP-based drug delivery. Nanodelivery has been an excellent way to treat cancer, including PC. It is easy to deliver the drugs simvastatin and docetaxel for prostate cancer through nanodelivery. It has both benefits and drawbacks, such as active tumor targeting, passive tumor accumulation, and transport across tissue barriers (such as toxicity and organ damage). Even though many NP-based drug delivery systems have been used for a long time to treat PC in vitro or in vivo, each system and method has some problems. With an active targeting strategy, the prostate can be targeted from a loco regional route that can deliver the most drugs to the tumor.

## Figures and Tables

**Table 1:** List of investigational drugs under clinical trials for prostate cancer treatment (source: ClinicalTrials.gov).

Interventions	Conditions	Characteristics		Population		NCT Number	Status
		Phases	Study Type	Age	Sex		
Diagnostic test: Blood sample	PC	Not Applicable	IV	40 years or above	Male	NCT04556916	Recruiting
Drug: ODM- Drug: ADT	PC	2	IV	18 years or above	Male	NCT02972060	Active, not recruiting
Drug: cholecalciferol Other: placebo	PC	2	IV	19 years to 90 years	Male	NCT02726113	Completed
Diagnostic test: 18FPSMA	PC	1	IV	40 years to 70 years	Male	NCT03558711	Completed
Drug: Leuprolide	PC	4	IV	18 years or above	Male	NCT03035032	Completed
Diagnostic test: [18F] PSMA-11	PC	2	IV	18 years or above	Male	NCT03573011	Completed
Drug: 18F-DCFPyL PET	Prostate Cancer Prostate Neoplasm	Early Phase 1	IV	18 years or above	Male	NCT03232164	Recruiting
Dietary supplement: green tea capsules. Behavioral: Green tea drink. Other: Green tea placebo capsules. Other: Lycopene placebo capsules. Other: Tomato-rich diet. Dietary supplement: Lycopene capsules	PC	2 and 3	IV	50 Years to 69 Years	Male	NCT01105338	Completed
Drug: High-dose testosterone	Metastatic Prostate Cancer	2	IV	18 years or above	Male	NCT05011383	Recruiting
Drug: 18F-DCFBC	PC	1 and 2	IV	18 years to 90 years	Male	NCT01496157	Completed
Drug: ELIGARD	PC	2	IV	18 years or above	Male	NCT02274779	Active, not recruiting
Radiation: Radiation therapy	PC	2	IV	18 years to 90 years	Male	NCT04984343	Recruiting
Drug: Enzalutamide Drug: Leuprolide acetate Radiation: radiation	PC	2	IV	18 years to 90 years	Male	NCT02064582	Completed
Drug: PD1-PSMACART cells	PC	1	IV	18 years to 75 years	Male	NCT04768608	Completed
Drug: BR55	PC	1 and 2	IV	50 years to 70 years	Male	NCT02142608	Completed

## Data Availability

Data can be made available on request to correspondence authors.
